# *E*-Configuration Improves Antioxidant and Cytoprotective Capacities of Resveratrols

**DOI:** 10.3390/molecules23071790

**Published:** 2018-07-20

**Authors:** Jian Lin, Xican Li, Ban Chen, Gang Wei, Dongfeng Chen

**Affiliations:** 1 School of Basic Medical Science, Guangzhou University of Chinese Medicine, Waihuan East Road No. 232, Guangzhou Higher Education Mega Center, Guangzhou 510006, China; linjianchn@outlook.com; 2 The Research Center of Basic Integrative Medicine, Guangzhou University of Chinese Medicine, Waihuan East Road No. 232, Guangzhou Higher Education Mega Center, Guangzhou 510006, China; 3 School of Biomedical Sciences, Monash University, Melbourne Victoria 3001, Australia; 4 School of Chinese Herbal Medicine, Guangzhou University of Chinese Medicine, Waihuan East Road No. 232, Guangzhou Higher Education Mega Center, Guangzhou 510006, China; imchenban@foxmail.com (B.C.); weigang@gzucm.edu.cn (G.W.); 5 Innovative Research & Development Laboratory of TCM, Guangzhou University of Chinese Medicine, Waihuan East Road No. 232, Guangzhou Higher Education Mega Center, Guangzhou 510006, China

**Keywords:** *Z*/*E*-configuration, antioxidant, *Z*-resveratrol, geometrical configuration, dihedral angle, bmMSCs, cytoprotective effect

## Abstract

The antioxidant and cytoprotective capacities of *E*-resveratrol and *Z*-resveratrol were compared using chemical and cellular assays. Chemical assays revealed that the two isomers were dose-dependently active in •O_2_^−^-scavenging, ferric reducing antioxidant power (FRAP), Cu^2+^-reducing antioxidant capacity (CUPRAC), 2-phenyl-4,4,5,5-tetramethylimidazoline-1-oxyl 3-oxide radical (PTIO•)-scavenging (pH 7.4 and pH 4.5), and 1,1-diphenyl-2-picryl-hydrazyl (DPPH•)-scavenging assays. The cellular assay indicated that the two isomers could also increase cell viabilities. However, quantitative analyses suggested that *E*-resveratrol exhibited stronger effects than *Z*-resveratrol in all chemical and cellular assays. Finally, the conformations of *E*-resveratrol and *Z*-resveratrol were analyzed. It can be concluded that both *E*-resveratrol and *Z*-resveratrol can promote redox-related pathways to exhibit antioxidant action and consequently protect bone marrow-derived mesenchymal stem cells (bmMSCs) from oxidative damage. These pathways include electron transfer (ET) and H^+^-transfer, and likely include hydrogen atom transfer (HAT). The *E*-configuration, however, improves antioxidant and cytoprotective capacities of resveratrols. The detrimental effect of the *Z*-configuration may be attributed to the non-planar preferential conformation, where two dihedral angles block the extension of the conjugative system.

## 1. Introduction

The carbon-carbon double bond (C=C) occurs widely in phenolic antioxidants, including phenolic phenylpropanoid [1], cinnamic acid derivatives [2], oligostilbenes [3], chalcones [4], flavonoids [5], and flavonoid glycosides [6]. The C=C position has recently been suggested to alter antioxidant levels of phenolic antioxidants via conjugation [3,7]. As a planar covalent bond, C=C may also present different geometrical configurations. These geometrical configurations may bring about different relative positions and interactions between functional groups. These interactions are hypothesized to affect the antioxidant capacity of phenolic antioxidants. However, no relevant studies regarding the effect of geometrical configuration on antioxidant capacity have been reported until now. 

**Figure 1:**
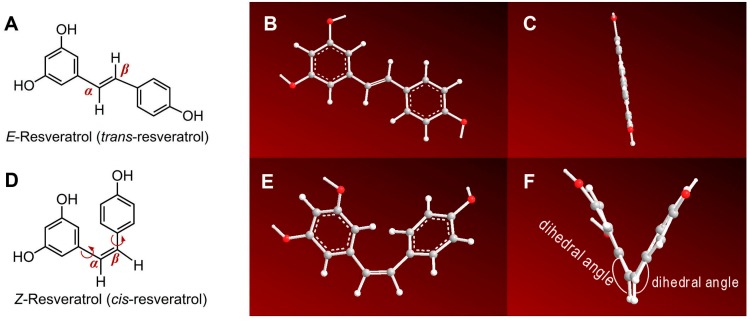
The configuration formula and molecular models of two resveratrols: (**A**) configuration formula of *E*-resveratrol; (**B**) preferential conformation-based ball-and-stick model of *E*-resveratrol (front view); (**C**) preferential conformation-based ball-and-stick model of *E*-resveratrol (right view); (**D**) configuration formula of *Z*-resveratrol; (**E**) preferential conformation-based ball-and-stick model of *Z*-resveratrol (front view); (**F**) preferential conformation-based ball-and-stick model of *Z*-resveratrol (right view). (The preferential conformation was created using ChemBioOffice 2014/Chem3D Pro.)

Due to the central role of C=C in the stilbene core, stilbene compounds were selected as the focus of this study. The most typical stilbene compound is *trans*-resveratrol (Figure 1A), which is widely found in red wine and many Chinese herbal medicines [8,9]. A great number of studies indicated that *trans*-resveratrol had beneficial effects on neurological diseases (e.g., Alzheimer disease [10], depressive symptoms [11]) and chronic diseases (e.g., hepatitis) [12,13]. The latest studies however suggested that it could also exhibit versatile activities, such as anti-cancer [14], anti-obesity [15], and anti-aging effects [16]. These effects are closely associated with antioxidant effects, in accordance with free radical biology and medicine [17]. Correspondingly, many studies focusing on the antioxidant effects and mechanisms of *trans*-resveratrol and the analogs have been reported [18,19,20]. Therefore, *trans*-resveratrol is considered to be an effective stilbene antioxidant. Usually, the so-called “resveratrol” in the literature refers to *trans*-resveratrol [13].

Strictly, the configuration of trans-resveratrol should be termed as the *E*-configuration, according to International Union of Pure and Applied Chemistry Rules [21]. Correspondingly, its isomer *cis*-resveratrol (Figure 1B) should be denominated *Z*-resveratrol. Hence, the two isomers *E*-resveratrol and *Z*-resveratrol provide an ideal pairing to research the effect of geometrical *Z/E*-configuration on the antioxidant properties of phenolics.

However, the issue has so far only been examined by computational chemists. In 2010, Mikulski and colleagues used computational chemistry to compare the radical scavenging activities of seven stilbenes (especially *Z*-resveratrol and *E*-resveratrol) in gas phase and water environments. All studied stilbenes had a higher hydrogen atom transfer (HAT) potential in the gas phase than in aqueous medium, where they were sensitive to electron transfer (ET) [22]. The study is obviously severely limited, because of the absence of experimental evidence from chemistry and biology.

The reason why previous studies have been limited to computational chemistry, with no experimental chemistry, may be the fact that *Z*-resveratrol is chemically unstable. The C=C in stilbenes generally displays the *E*-configuration [23]. As seen in Figure 1B, in the *Z*-resveratrol molecule, two benzo-rings are arrayed on one side of the C=C; this is therefore a crowded configuration with high molecular energy. Thus, it is difficult to prepare *Z*-resveratrol through common synthetic approaches. It is not available for commercial purchase. Compared to *E*-resveratrol, *Z*-resveratrol (Figure 1B) has received much less interest from scientists, possibly due to its difficulty of preparation.

In the present study, authentic samples of *Z*-resveratrol, along with *E*-resveratrol were comparatively characterized using chemical and cellular assays. Chemical assays included •O_2_^−^-scavenging, ferric reducing antioxidant power (FRAP), Cu^2+^-reducing antioxidant capacity (CUPRAC), 2-phenyl-4,4,5,5-tetramethylimidazoline-1-oxyl 3-oxide radical (PTIO•)-scavenging (pH 7.4 and pH 4.5), and 1,1-diphenyl-2-picryl-hydrazl (DPPH•)-scavenging assays. In the biological assay, bone marrow-derived mesenchymal stem cells (bmMSCs) acted as the cellular model. Due to the crucial role of bmMSCs in transplant engineering and beneficial effect of resveratrol in Alzheimer’s disease [24], the study will provide new information on candidate antioxidant treatments for bmMSCs transplant engineering (particularly for the treatment of Alzheimer’s disease). It will also provide definitive information on the role of the *Z*/*E* configuration in phenolic antioxidants. These conclusions will aid the understanding of other similar phenolic antioxidants, including stilbenes (e.g., *E*-3,5-dihydroxystilbene and *Z*-3,5-dihydroxystilbene, Suppl. 1), and 2-phenyl-benzofuran-stilbene hybrids (e.g., maximol A and maximol B, Suppl. 2).

## 2. Results and Discussion

To provide supporting evidence in an experimental chemical system, the interaction of each resveratrol with reactive oxygen species (ROS) was explored using the pyrogallol autoxidation assay, a superoxide (•O_2_^−^) scavenging assay improved by our team [25]. The inhibition of pyrogallol autoxidation can characterize the •O_2_^−^ -scavenging levels of an antioxidant. As seen in Suppl. 3, the •O_2_^−^-scavenging percentages of both isomers increased in a dose-dependent manner. This suggests that the two resveratrols had ROS-scavenging potential.

As one form of ROS-scavenging, •O_2_^−^-scavenging has been suggested to be involved in several antioxidant pathways, including ET [26], H^+^-transfer [27], HAT [27], and proton-coupled electron transfer (PCET) [28,29]. These pathways are all fundamentally involved in ET and H^+^-transfer. The differences between them lie in the sequence of steps and their coordination. ET and H^+^-transfer simultaneously occur as a unit in the HAT pathway. However, ET and H^+^-transfer take place separately and subsequently in the PCET [30] and sequential electron proton transfer (SEPT) [31] pathways. However, the final outcome is identical, i.e., an antioxidant donates an electron and a proton to •O_2_^−^. H^+^-transfer can cause protonated •O_2_^−^, namely hydroperoxide (HO_2_•) [27,32]. HO_2_• however has a much higher oxidation potential than •O_2_^−^. ET can convert •O_2_^−^ into the stable O_2_ molecule to lower the redox potential (E_0_’ = 0.31 V, pH 7.0) [33]. Thus, ET plays a key role in •O_2_^−^- and ROS-scavenging. The fact that two resveratrols can effectively scavenge the •O_2_^−^-radical may indicate an ET potential in their antioxidant reactions.

To test the ET potential, the two resveratrols were further assessed by FRAP assay, a Fe^3+^-reducing reaction under acidic conditions (pH 3.6) [34,35]. Such acidic conditions are believed to effectively suppress the H^+^-transfer from phenolics with weak acidity and thus the FRAP assay has been considered a purely ET reaction [36]. As shown in Suppl. 3, the FRAP values of both isomers increased in a strong dose-dependent manner, indicating that they had potential ET activity. In addition, at pH 7.4, both isomers were observed to increase the percentage of Cu^2+^-reducing in a strongly dose-dependent manner. This implies that, at physiological pH, both isomers still possessed potential ET activity.

The two metal-reducing reactions (Fe^3+^- and Cu^2+^-reducing) are not identical radical-scavenging reactions and occur via distinct mechanisms. In order to provide evidence of ET in these radical-scavenging processes, the two isomers were further analyzed using a PTIO•-scavenging assay, a new method established by our team [37]. The PTIO•-scavenging assay can be conducted at various pH values in aqueous media. However, in aqueous buffer with pH ≤ 5.0, the PTIO•-scavenging reaction was confirmed by cyclic voltammetry to be a pure ET process [37,38]. The observations that both isomers had elevated PTIO•-scavenging ability at pH 4.5 indicated that they were able to exhibit potential ET activity in the radical-scavenging reactions.

Unlike at pH 4.5, PTIO•-scavenging at pH 7.4 was regarded as being involved in H^+^-transfer. Through H^+^-transfer, PTIO• was transformed into [PTIO-H]^+^ and the process could be monitored by HPLC-MS [37]. The findings that two isomers could effectively scavenge PTIO• at physiological pH 7.4, suggested that the two isomers might also undergo H^+^-transfer to scavenge radicals. The involvement of the H^+^-transfer pathway obviously accelerates the radical-scavenging reaction, thus, their IC_50_ values at pH 7.4 always were lower than those at pH 4.5 (Table 1).

As proposed by the computational chemistry [22], the two isomers preferentially underwent HAT (donating a hydrogen atom) to exert antioxidant activity in the gas phase. In our experiment with authentic samples, it was observed that the two resveratrols concentration-dependently scavenged DPPH• radical (Suppl. 3); and their IC_50_ values were close to that of Trolox, a well-characterized antioxidant (Table 1). DPPH•-scavenging has been reported to include at least one HAT pathway, although there are other antioxidant pathways involved, such as proton-coupled electron transfer (PCET), sequential-proton-loss-electron-transfer (SPLET), single electron transfer (SET), and radical adduct formation (RAF) [39,40]. Thus, our findings provided experimental evidence of HAT pathway activity in two resveratrol isomers in chemical solution. To some extent, the evidence of DPPH•-scavenging can partly support the aforementioned H^+^-transfer and ET reactions, because HAT is regarded as a synergetic and simultaneous process of H^+^-transfer *plus* ET. 

In short, both *E*-resveratrol and *Z*-resveratrol may act via similar pathways to exert their antioxidant actions. These pathways at least comprise H^+^-transfer and ET and possibly HAT. Essentially, these pathways are based on redox reactions. 

However, the two resveratrols were quite different in their relative antioxidant levels. As seen in Table 1, in all five antioxidant assays, *E*-resveratrol showed lower IC_50_ values than *Z*-resveratrol. This clearly suggested that *E*-resveratrol was a stronger antioxidant, compared to *Z*-resveratrol. Undoubtedly, such difference can be attributed to the geometrical configurations, i.e., *E*/*Z*-configurations.

Seemingly, the *E*/*Z*-configuration merely alters the geometrical sites of two phenyl groups (*α*-phenyl and *β*-phenyl). In fact, such geometrical configuration can further cause differences in the molecule’s preferential conformations. In the *E*-configuration, two phenyl groups can stably occupy the two sides of the C=C, thus they share a plane (Figure 1B,C). By comparison, in the *Z*-configuration, two phenyl groups cannot stably occupy one side of the C=C, because of the crowding; *α*-phenyl and *β*-phenyl have to rotate their two σ bonds to reduce the crowding as much as possible (Figure 1D–E). Thus, there are two dihedral angles, between the α-phenyl plane and the *α*, *β*-C=C plane and between the *β*-phenyl plane and the planar *α*, *β*-C=C plane (Figure 1F). The whole *Z*-resveratrol molecule however is not on one plane. The existence of the dihedral angle effectively reduced the crowding of the two phenyl groups by introducing extensive steric hindrance. Therefore, the preferential conformations of *Z*-resveratrol and *E*-resveratrol are different; however, they are both generally stable. Our conformational analysis based on ChemBioOffice 2014/Chem3D Pro software (PerkinElmer, Guangzhou, China) was further supported by the computational chemistry, where the two dihedral angles in *Z*-resveratrol (Figure 1F) were calculated to be 39.22° and 27.43°; while *E*-resveratrol has no dihedral angles. The molecular energies of both isomers were similarly calculated as −766.571045 hartree (approximately −25.2 eV) [22].

**Figure 2:**
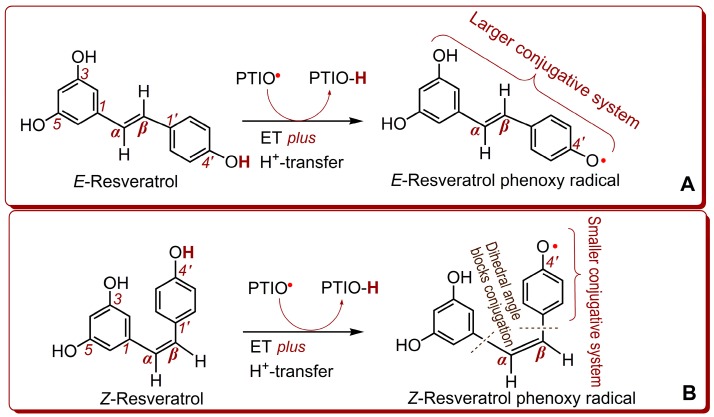
The proposed reactions of *E*-resveratrol (**A**) and *Z*-resveratrol (**B**) with PTIO radical via ET *plus* H^+^-transfer pathways.

It has been suggested that the antioxidant capacity of phenolics usually depends on the stability of their product after oxidation by free radicals (especially ROS) [41]. Irrespective of which pathway mediated the reaction, the two resveratrols oxidized by ROS can similarly produce a phenoxy radical, which may further be transferred into a semi-quinone (or even a quinone) form. Thus, the stability of the phenoxy radical may be responsible for the antioxidant capacity of a phenolic reactant. One mole of *E*-resveratrol reacts with 1 mol PTIO• via the H^+^-transfer *plus* ET pathways to generate a resveratrol phenoxy radical at the 4’-position [42]. As illustrated in Figure 2A, this phenoxy radical had a larger conjugative system. By comparison, 1 mol *Z*-resveratrol reacts similarly with 1 mol PTIO• to give a phenoxy radical at the 4’-position (Figure 2B). However, the *Z*-resveratrol phenoxy radical is less stable than the *E*-resveratrol phenoxy radical. This is because the two dihedral angles block the extension of conjugation in the *Z*-resveratrol phenoxy radical. The reduced and scattered conjugation dictates the instability of the *Z*-resveratrol phenoxy radical, and, therefore, the weaker antioxidant capacity of *Z*-resveratrol as a reactant.

**Figure 3:**
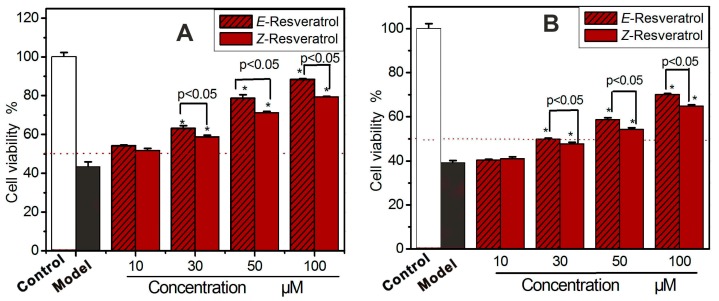
Cytoprotective effects of two resveratrols on oxidatively damaged bmMSCs: (**A**) bmMSCs were damaged by H_2_O_2_; (**B**) bmMSCs were damaged by Fenton reagent (i.e., addition of 100 μM FeCl_2_ followed by 50 μM H_2_O_2_). The control group was cultured in medium only, while the model group was treated with H_2_O_2_ (or Fenton reagent). The resveratrol group was damaged by H_2_O_2_ (or Fenton reagent) followed by *E*- or *Z*-resveratrol. Each value is expressed as the mean ± SD, *n* = 3; * Significant difference *vs* the model group, *p* < 0.05. Cell viability was assessed using the MTT method. bmMSCs, bone marrow-derived mesenchymal stem cells; MTT, methyl thiazolyl tetrazolium.

To obtain biological evidence, the two resveratrols were incubated with bmMSCs damaged by H_2_O_2_ and Fenton reagent (an •OH radical generator). The survival of bmMSCs was characterized by MTT assay [43]. As seen in Figure 3A,B, the two resveratrols could concentration-dependently increase the survival of bmMSCs at 10–100 μM. Hence, both the resveratrols could resist not only H_2_O_2_, but also the •OH radical, to protect bmMSCs from oxidative damage. This obviously supported the redox-related antioxidant pathways proposed above. However, as seen in Figure 3, *E*-resveratrol displayed a better cytoprotective effect than *Z*-resveratrol. The difference in cytoprotective effects is consistent with the difference in cytoprotective effect antioxidant effects. 

In summary, both chemical and cellular evidence suggested that *E*-resveratrol had higher antioxidant and cytoprotective effects than *Z*-resveratrol. As the sole chemical difference between two resveratrols is their geometrical configuration, the geometrical configuration is likely to be fully responsible for these differences. Thus, it can be inferred that the *E*-configuration improved antioxidant and cytoprotective capacities of resveratrols. The reason for *Z*-resveratrol being inferior to *E*-resveratrol as an antioxidant cytoprotector is that the *Z*-configuration makes the functional group crowded, especially for such large groups. The crowded arrangement can further cause the preferential conformation to be a dihedral angle, which can destroy the molecular planarity. Non-planarity can block the formation of a larger conjugative system (especially π-π conjugation). This greatly reduced the delocalization effect of the phenoxy radical and the phenoxy radical as an oxidized product correspondingly became unstable. The phenolic reactant was therefore inactivated in radical-scavenging reactions. These findings provide new understanding of the antioxidant effects of resveratrols and other phenolics, and will also help synthetic chemists to design more effective C=C-containing antioxidant candidates for bmMSC transplantation therapy [44,45]. In addition, these findings can explain the reason why *E*-resveratrol can widely occur in nature, and has versatile bioactivities [10,11,12,13,15,16].

## 3. Materials and Methods

### 3.1. Animals and Chemicals

Sprague-Dawley (SD) rats at 4 weeks of age were obtained from the animal center of the Guangzhou University of Chinese Medicine. The protocol of this experiment was performed under the supervision of the Institutional Animal Ethics Committee at the Guangzhou University of Chinese Medicine. Other reagents were of analytical grade. *E*-Resveratrol (CAS 501-36-0, C_14_H_12_O_3_, M.W. 228.2, purity 97%) was obtained from BioBioPha Co., Ltd. (Kunming, China, Suppl. 4); *Z*-resveratrol (CAS 61434-67-1, C_14_H_12_O_3_, M.W. 228.2, purity 97%, Suppl. 4) was kindly donated by the School of Biomedical Sciences, Monash University (Melbourne, Australia). 1,1-Diphenyl-2-picrylhydrazyl radical (DPPH•), (±)-6-hydroxyl-2,5,7,8-tetramethylchromane-2-carboxylic acid (Trolox), pyrogallol, and 2,9-dimethyl-1,10-phenanthroline (neocuproine) were purchased from Sigma-Aldrich Shanghai Trading Co. (Shanghai, China). 2,4,6-Tripyridyltriazine (TPTZ), pyrogallol, and 3-(4,5-dimethylthiazol-2-yl)-2,5-diphenyltetrazolium bromide (MTT) were obtained from Sigma-Aldrich Shanghai Trading Co. Dulbecco’s modified Eagle’s medium (DMEM) and fetal bovine serum (FBS) were purchased from Gibco (Grand Island, NY, USA). CD44 and Proteinase K were purchased from Wuhan Boster Co., Ltd. (Wuhan, China). All other reagents were of analytical grade.

### 3.2. Superoxide Anion (•O_2_^−^) Scavenging Assay

Superoxide anion (•O_2_^−^) inhibiting activity was measured using a pyrogallol autooxidation method that was previously improved by our laboratory [41]. Briefly, the sample was dissolved in methanol at 1 mg/mL. The sample solution (*x* = 50–250 μL) was mixed with Tris-HCl buffer (980-*x* μL, 0.05 M, pH 7.4) containing EDTA (1 mM). After 20 μL pyrogallol (60 mM in 1 mM HCl) was added, the mixture was vigorously shaken at room temperature. The absorbance of the mixture was measured (Unico 2100, Shanghai, China) at 325 nm every 30 s for 5 min. Tris-HCl buffer was used as a blank. The •O_2_^−^ inhibiting ability was calculated as follows:

$$\mathrm{Inhibition}\;\%=\frac{\left(\frac{A_{325nm,control}}{T}-\frac{A_{325nm,sample}}{T}\right)}{\left(\frac{A_{325nm,control}}{T}\right)}\times 100\%$$

where Δ*A*_325nm_, _control_ is the increment in the absorbance at 325 nm (*A*_325nm_) of the mixture without the sample and Δ*A*_325nm_, _sample_ is the increment in *A*_325nm_ of the mixture including the sample; *T* = 5 min.

### 3.3. Ferric-Reducing Antioxidant Power (FRAP) Assay

The FRAP assay was adapted from Benzie and Strain [46]. Briefly, the FRAP reagent was freshly prepared by mixing 10 mM TPTZ, 20 mM FeCl_3_, and 0.25 M pH 3.6 acetate buffer at 1:1:10 (volume ratio). The test sample (*x* = 2–10 μL, 0.5 mg/mL) was added to (20−*x*) μL of 95% ethanol followed by 80 μL of FRAP reagent. The absorbance was measured at 595 nm after a 30 min incubation at ambient temperatures using distilled water as the blank. The relative reducing power of the sample compared to the maximum absorbance was calculated using the following formula:

$$\mathrm{Relative}\;\mathrm{reducing}\;\mathrm{effect}\;\%=\frac{A-A_{min}}{A_{max}-A_{min}}\times 100\%$$

where *A*_max_ is the maximum absorbance at 595 nm and *A*_min_ is the minimum absorbance in the test. *A* is the absorbance of the sample.

### 3.4. Cupric Ions (Cu^2+^) Reducing Antioxidant Capacity (CUPRAC) Assay

The cupric ion reducing antioxidant capacity (CUPRAC) assay was determined based on the method proposed by Apak et al. [47], with small modifications as presented in the literature of Tian [48]. Twelve microliters CuSO_4_ solution (0.01 M) and 12 μL ethanolic neocuproine solution (7.5 × 10^−3^ M) were added to a 96-well and mixed with different concentrations of samples (3–15 μg/mL). The total volume was then adjusted to 100 μL with a CH_3_COONH_4_ buffer solution (0.1 M), and mixed again to homogenize the solution. The mixture was maintained at room temperature for 30 min, and the absorbance was measured at 450 nm on a microplate reader (Multiskan FC, Thermo Scientific, Shanghai, China). The relative reducing power of the sample was calculated using the formula in Section 3.3.

### 3.5. PTIO•-Scavenging Assays

The PTIO•-scavenging assays (at pH 4.5 or pH 7.4) were conducted based on our previously described method [32]. In brief, the test sample solution (*x* = 2–10 μL, 1 mg/mL) was added to (20−*x*) μL of 95% ethanol, followed by 80 μL of an aqueous PTIO• solution. The aqueous PTIO• solution was prepared using a phosphate-butter solution (0.1 mM, pH 4.5 or pH 7.4). The mixture was maintained at 37 °C for 2 h, and the absorbance was then measured at 560 nm using the aforementioned microplate reader. The PTIO• inhibition percentage was calculated as follows:

$$\mathrm{Inhibition}\;\%=\frac{A_{0}-A}{A_{0}}\times 100\%$$

where *A*_0_ is the absorbance of the control without the sample and *A* is the absorbance of the reaction mixture with the sample.

### 3.6. DPPH•-Scavenging Assay

DPPH• radical scavenging activity was determined as previously described [49]. Briefly, 80 μL of DPPH• solution (0.1 mol/L) was mixed with methanolic sample solutions with the indicated concentration (0.05 mg/mL, 5–25 μL). The mixture was maintained at room temperature for 30 min and the absorbance was measured at 519 nm on a microplate reader. The percentage of DPPH• scavenging activity was calculated using the equation described in Section 3.5.

### 3.7. Protective Effect Against Fenton-induced Damage to bmMSCs (MTT assay)

**Figure 4:**
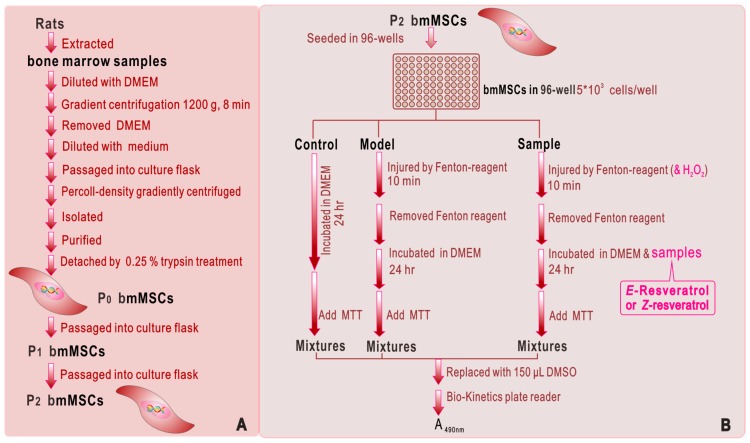
Experimental procedures for the preparation and culture of bmMSCs (**A**) and for the MTT assay (**B**). Each test was repeated in five independent wells. Fenton reagent: FeCl_2_ (100 μM) followed by H_2_O_2_ (50 μM); MTT: 5 mg/mL in PBS, 20 μL; PE-1420 Bio-Kinetics reader: Bio-Kinetics Corporation, Sioux Center, IA, USA.

bmMSCs culture was carried out according to our previous report [50] with slight modifications. bmMSCs at passage 3 were analyzed for cell homogeneity based on CD44 expression by flow cytometry (Figure 4A). The protective effect of resveratrols against oxidative damage of bmMSCs was evaluated using the MTT assay [51]. The experimental protocol is briefly illustrated in Figure 4B.

### 3.8. Statistical Analysis

Each experiment was performed in triplicate and the data were recorded as mean ± SD (standard deviation). The dose-response curves were plotted using Origin 6.0 professional software (OriginLab, Northampton, MA, USA). The IC_50_ value was defined as the final concentration of 50% radical inhibition (or relative reducing power) [52]. It was calculated by linear regression analysis and expressed as the mean ± SD (*n* = 3). The linear regression was analyzed using Origin 6.0. Determination of significant differences between the mean IC_50_ values was performed using one-way ANOVA and the *t*-test. The analysis was performed using SPSS software 13.0 (SPSS Inc., Chicago, IL, USA) for windows. *p* < 0.05 was considered to be statistically significant.

## 4. Conclusions

Both *E*-resveratrol and *Z*-resveratrol can induce redox-related pathways to exhibit antioxidant action. These pathways include ET and H^+^-transfer and may include HAT. Thus, antioxidant resveratrols can protect bmMSCs from oxidative damage. However, *E*-resveratrol always exhibited stronger antioxidant and cytoprotective effects than *Z*-resveratrol. The difference can be attributed to the existence of two dihedral angles, which destroy the molecular planarity and thus block the extension of the conjugative system. In other words, the *Z*-configuration plays a detrimental role and the *E*-configuration improves antioxidant and cytoprotective capacities of resveratrols.

## Figures and Tables

**Table 1:** The IC_50_ values of *E*-resveratrol and *Z*-resveratrol in various antioxidant assays.

Assays	*E*-Resveratrol μM	*Z*-Resveratrol μM	Trolox μM
•O_2_^−^-scavenging	345.3 ± 5.5 ^a^	448.2 ± 4.9 ^b^	507.1 ± 30.4 ^c^
FRAP	20.7 ± 0.7 ^a^	22.1 ± 0.9 ^a^	28.9 ± 0.8 ^c^
Cu^2+^-reducing	24.5 ± 0.3 ^a^	30.7 ± 0.4 ^b^	31.9 ± 0.3 ^c^
PTIO•-scavenging (pH 4.5)	395.9 ± 3.9 ^a, B^	436.3 ± 4.9 ^b, B^	206.9 ± 6.5 ^a^
PTIO•-scavenging (pH 7.4)	157.7 ± 3.2 ^a, A^	198.2 ± 0.9 ^b, A^	383.8 ± 8.8 ^c^
DPPH•-scavenging	24.3 ± 0.1 ^a^	29.3 ± 0.2 ^c^	25.6 ± 0.2 ^b^

The IC_50_ value was defined as the lowest concentration with 50% radical inhibition or relative reducing power, calculated by linear regression analysis, and expressed as the mean ± SD (*n* = 3). The linear regression was analyzed by Origin 6.0 professional software. The IC_50_ values with different superscripts (a, b, or c) in the same row and the same column (A or B) are significantly different (*p* < 0.05). Trolox was the positive control.

## Supplementary Materials

The following are available online: Suppl. 1, The configuration formula of *E*-3,5-dihydroxystilbenen and *Z*-3,5-dihydroxystilbenen; Suppl. 2, The configuration formula of maximol A and maximol B; Suppl. 3, Dose-response curves; Suppl. 4 The certificate of analysis *E*-resveratrol and *Z*-resveratrol.

## Abbreviations

The following abbreviations are used in this manuscript:

bmMSCs	Bone marrow-derived mesenchymal stem cells
CUPRAC	Cu^2+^-reducing antioxidant capacity
DPPH•	1,1-Diphenyl-2-picrylhydrazyl
DMEM	Dulbecco’s modified Eagle’s medium
ET	Electron-transfer
FBS	Fetal bovine serum
FRAP	Ferric reducing antioxidant power
HAT	Hydrogen atom transfer
MTT	Methylthiazolyltetrazolium. *Or*: 3-(4,5-dimethylthiazol-2-yl)-2,5-diphenyltetrazolium bromide
PCET	Proton-coupled electron transfer
PTIO•	2-Phenyl-4,5-tetramethylimidazoline-1-oxyl 3-oxide radical
RAF	Radical-adduct-formation
ROS	Reactive oxygen species
SD	Standard deviation
SPLET	Sequential-proton-loss-electron-transfer
SEPT	Sequential electron proton transfer
SET	Single electron transfer
TPTZ	2,4,6-tris(2-Pyridyl-s-triazine)
Trolox	(±)-6-Hydroxyl-2,5,7,8-tetramethlychromane-2-carboxylic acid
