# Supplementary material for: E-Configuration Improves Antioxidant and Cytoprotective Capacities of Resveratrols
Source: Molecules. 2018 Jul 20;23(7):1790. doi: 10.3390/molecules23071790 (PMC6100583; doi:10.3390/molecules23071790)
Supplement: Supplementary file 1 [file molecules-23-01790-s001.zip › molecules-327814-SI/Suppl. 3 Dose response curves.pdf]

Supplementary Materials-3 Dose response curves

E-Configuration Improves Antioxidant and Cytoprotective Capacities of Resveratrols

Jian Lin [1,2], Xican Li [3,4], Ban Chen [3,4], Gang Wei [4,5], and Dongfeng Chen [1,2,*]

[1] School of Basic Medical Science, Guangzhou University of Chinese Medicine, Waihuan East Road No. 232, Guangzhou Higher Education Mega Center, Guangzhou 510006, China; linjianchn@outlook.com (J.L.)

[2] The Research Center of Basic Integrative Medicine, Guangzhou University of Chinese Medicine, Waihuan East Road No. 232, Guangzhou Higher Education Mega Center, Guangzhou 510006, China;

[3] School of Biomedical Sciences, Monash University, Melbourne, Australia.

[4] School of Chinese Herbal Medicine, Guangzhou University of Chinese Medicine, Waihuan East Road No. 232, Guangzhou Higher Education Mega Center, Guangzhou 510006, China; imchenban@foxmail.com (B.C.)

[5] Innovative Research & Development Laboratory of TCM, Guangzhou University of Chinese Medicine, Waihuan East Road No. 232, Guangzhou Higher Education Mega Center, Guangzhou 510006, China;

* Correspondence: lixican@126.com or lixc@gzucm.edu.cn (X.L.); chen888@gzucm.edu.cn (D.C.); Tel.: +86-203-935-8076 (X.L.)

Note: This Supporting information provides the original data of Table 1 in the main text. All data underline are mentioned in **Table 1** in the main text.

1. *Superoxide anion (•O_2^-) scavenging assay*

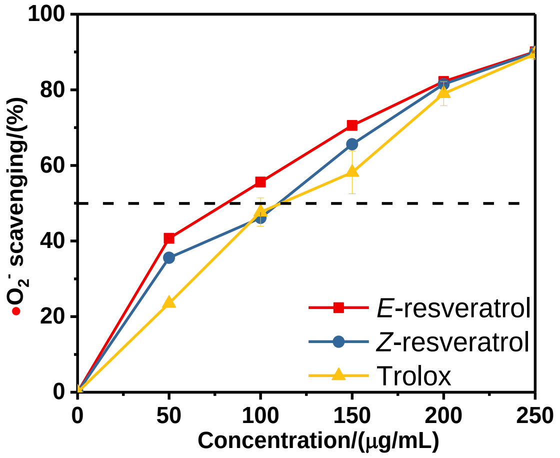

Fig. S3.1 The does response curves of *E*-resveratrol and *Z*-resveratrol and the positive control in •O_2^- scavenging assay.

Tab. S3.1 The comparison of IC_{50} values of *E*-resveratrol and *Z*-resveratrol and positive control in •O_2^- scavenging assay.

	Mean±SD μg/mL	Mean±SD μM
E-resveratrol	78.8±1.3	345.3±5.5[a]
Z-resveratrol	102.3.3±1.1	448.2±4.9[b]
Trolox	127.0±7.6	507.1±30.4[c]

IC_{50} value was defined as the concentration of 50% superoxide anion radical inhibition and calculated by linear regression which was analyzed by Origin 6.0 professional software. Means values with different superscripts in the same column are significantly different (p<0.05).

2. *Ferric-reducing antioxidant power (FRAP) assay (Fe^{3+}-Reducing)*

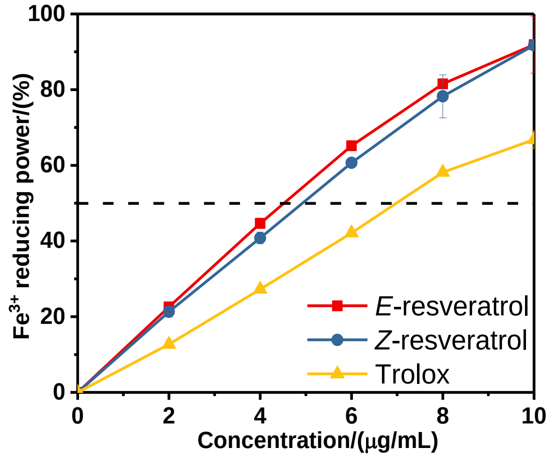

Fig. S3.2 The does response curves of *E*-resveratrol and *Z*-resveratrol and the positive control in FRAP assay.

Tab. S3.2 The comparison of IC_{50} values of *E*-resveratrol and *Z*-resveratrol and positive control in FRAP assay.

	Mean±SD μg/mL	Mean±SD μM
E-resveratrol	4.7±0.2	20.7±0.7[a]
Z-resveratrol	5.0±0.2	22.1±0.9[b]
Trolox	7.2±0.2	28.9±0.8[c]

IC_{50} value was defined as the concentration of 50% superoxide anion radical inhibition and calculated by linear regression which was analyzed by Origin 6.0 professional software. Means values with different superscripts in the same column are significantly different (p<0.05).

3. *Cupric ions (Cu^{2+}) reducing antioxidant capacity (CUPRAC) assay*

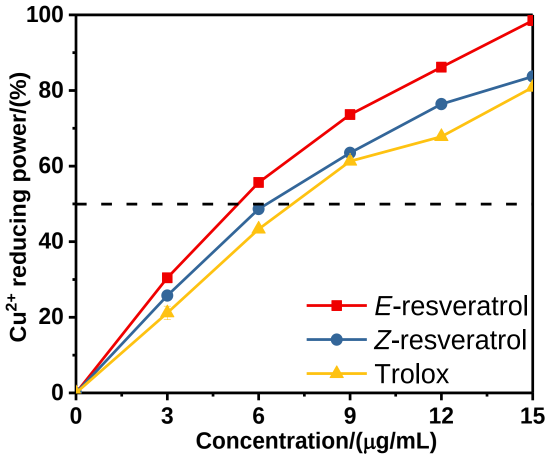

Fig. S3.3 The does response curves of *E*-resveratrol and *Z*-resveratrol and the positive control in Cu^{2+} reducing assay.

Tab. S3.3 The comparison of IC_{50} values of *E*-resveratrol and *Z*-resveratrol and positive control in Cu^{2+} reducing assay.

	Mean±SD μg/mL	Mean±SD μM
E-resveratrol	5.6±0.1	24.5±0.3[a]
Z-resveratrol	7.0±0.1	30.7±0.4[b]
Trolox	8.0±0.1	31.9±0.3[c]

IC_{50} value was defined as the concentration of 50% superoxide anion radical inhibition and calculated by linear regression which was analyzed by Origin 6.0 professional software. Means values with different superscripts in the same column are significantly different (p<0.05).

4. *PTIO•-scavenging assay (pH 4.5)*

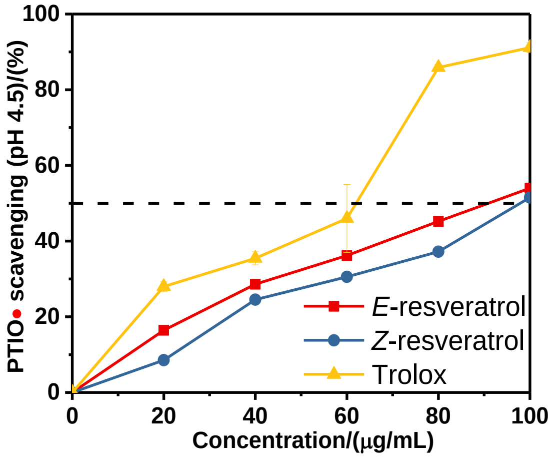

Fig. S3.4 The does response curves of *E*-resveratrol and *Z*-resveratrol and the positive control in PTIO•-scavenging assay (pH 4.5).

Tab. S3.4 The comparison of IC_{50} values of *E*-resveratrol and *Z*-resveratrol and positive control in PTIO•-scavenging assay (pH 4.5).

	Mean±SD μg/mL	Mean±SD μM
E-resveratrol	90.4±0.9	395.9±3.9[b]
Z-resveratrol	100.0±1.1	436.3±4.9[c]
Trolox	51.8±1.6	206.9±6.5[a]

IC_{50} value was defined as the concentration of 50% superoxide anion radical inhibition and calculated by linear regression which was analyzed by Origin 6.0 professional software. Means values with different superscripts in the same column are significantly different (p<0.05).

5. *PTIO•-scavenging assay (pH 7.4)*

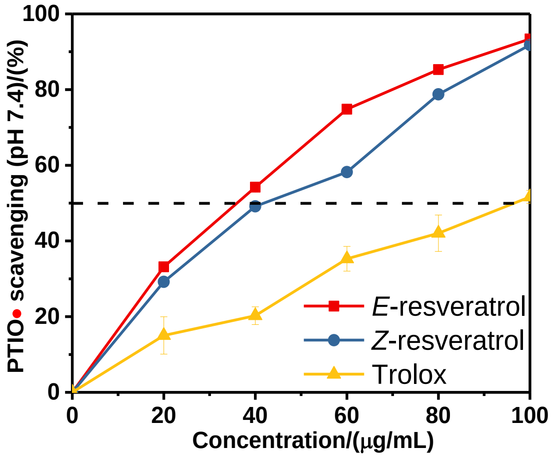

Fig. S3.5 The does response curves of *E*-resveratrol and *Z*-resveratrol and the positive control in PTIO•-scavenging assay (pH 7.4).

Tab. S3.5 The comparison of IC_{50} values of *E*-resveratrol and *Z*-resveratrol and positive control in PTIO•-scavenging assay (pH 7.4).

	Mean±SD<br>μg/mL	Mean±SD<br>μM
E-resveratrol	36.0±0.7	157.7±3.2[a]
Z-resveratrol	45.2±0.2	198.2±0.9[b]
Trolox	96.1±2.2	383.8±8.8[c]

IC_{50} value was defined as the concentration of 50% superoxide anion radical inhibition and calculated by linear regression which was analyzed by Origin 6.0 professional software. Means values with different superscripts in the same column are significantly different ($p<0.05$).

6. *DPPH•-scavenging assay*

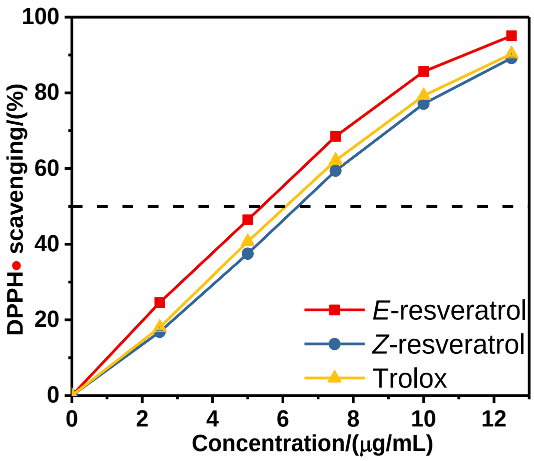

Fig. S3.6 The does response curves of *E*-resveratrol and *Z*-resveratrol and the positive control in DPPH•-scavenging assay.

Tab. S3.6 The comparison of IC_{50} values of *E*-resveratrol and *Z*-resveratrol and positive control in DPPH•-scavenging assay.

	Mean±SD<br>μg/mL	Mean±SD<br>μM
E-resveratrol	5.6±0.1	24.3±0.1[a]
Z-resveratrol	6.7±0.1	29.3±0.2[c]
Trolox	6.4±0.1	25.6±0.2[b]

IC_{50} value was defined as the concentration of 50% superoxide anion radical inhibition and calculated by linear regression which was analyzed by Origin 6.0 professional software. Means values with different superscripts in the same column are significantly different ($p<0.05$).
