# Supplementary material for: E-Configuration Improves Antioxidant and Cytoprotective Capacities of Resveratrols
Source: Molecules. 2018 Jul 20;23(7):1790. doi: 10.3390/molecules23071790 (PMC6100583; doi:10.3390/molecules23071790)
Supplement: Supplementary file 1 [file molecules-23-01790-s001.zip › molecules-327814-SI/Suppl. 4 Certificate of Analysis.pdf]

产品分析证书
Certificate of Analysis

中文名称：白藜芦醇

English Name：Resveratrol
别名(Alias):

产品编码(Cat. No.):BP1204
CAS Number: 501-36-0
分子式(M. F.): $C_{14}H_{12}O_3$
分子量(M. W.): 228.247

批号(Batch No.): PRF8091921
报告日期(Report date): 2017-09-19

Structure

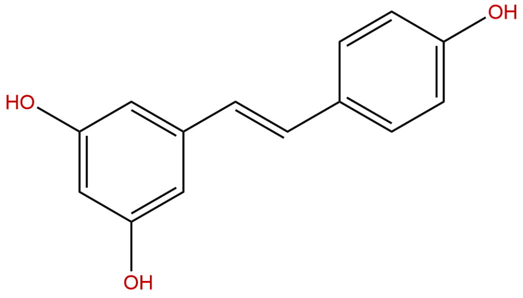

检验结果（Analytical result）:

检验项目（Test Item）	检验指标（Specifications）	检验结果（Results）
外观Appearance	Off-white powder	Off-white powder
纯度Purity (HPLC-DAD,306nm)*	≥98.0%	99.96%
质谱Mass	228.2±1	Conforms
核磁NMR	Comply with the structure	Conforms
水分Water Content	＜3.0%	1.17%

*** 色谱图见附件（Please find HPLC chromatography attached.）**

检测方法（Test Method）：Column: Thermo HyPURITY C18，4.6*150mm，3.0um; **Column temperature:** 30℃; **Detection Mode:** UV306nm; **Flow Rate:** 1.0ml/min; **Sample dissolution:** Methanol; **Mobile Phase: A,** 0.1% phosphoric acid in water **B**, Acetonitrile;**Gradient elution:** B, 20%-30%,15min.

贮存条件（Storage）:2~8℃, protected from light, keep package airproofed when not in use.

复测期（Retest date）:two years (2019-09-18) under conditions list above.

QC: Zhang Ling
Date: 2017-09-19

QA: Wu Qi
Date: 2017-09-19

备注(Remarks): The sample solutions should be prepared and used on the same day, it is the best preparing the solutions immediately before use. If the solutons have to be made up in advance, it should be made as aliquots in tightly sealed vials at less than -20℃. Generally, these might be useable for up to two weeks.
In case of quality issue, please contact us within 15 days after receipt of the product.

Chengdu Biopurify Phytochemicals Ltd. 成都普瑞法科技开发有限公司 质检专用章 Quality Approval Seal

Tel：+86-28-82633397 Fax：+86-28-82633165
http://www.phytopurify.com Email： sales@biopurify.com biopurify@gmail.com

SAMPLE INFORMATION

Sample Name:	Resveratrol	Acquired By:	System
Sample Type:	Standard	Sample Set Name:	
Vial:	32	Acq. Method Set:	Resveratrol
Injection #:	1	Processing Method:	Samples
Injection Volume:	10.00 ul	Channel Name:	306.0nm
Run Time:	25.0 Minutes	Proc. Chnl. Descr.:	PDA 306.0 nm
Date Acquired:	2017-9-19 11:12:01 CST		
Date Processed:	2017-9-19 12:36:57 CST		

Auto-Scaled Chromatogram

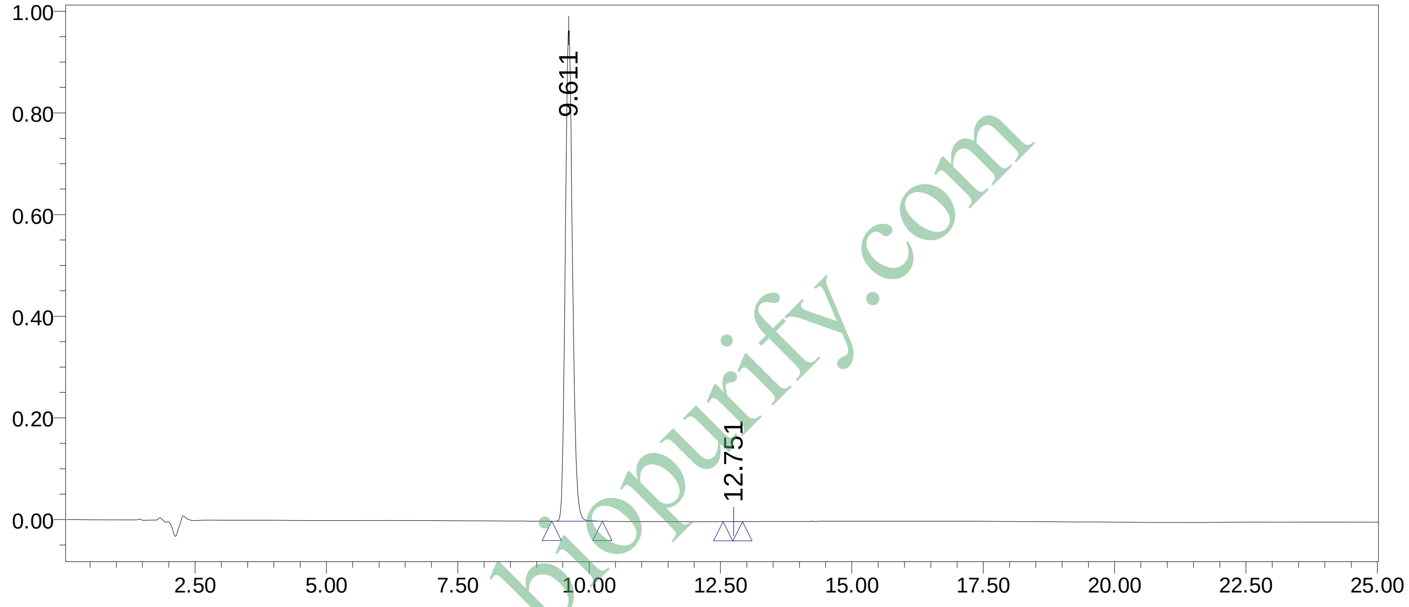

www.biopurify.com

Peak Results

	RT	Area	% Area	USP Plate Count	USP Resolution
1	9.611	9192525	99.96	23798.00	
2	12.751	3419	0.04	37980.93	11.75

Certificate of Analysis

1. Sample information

Chemical name: Cis-resveratrol / (Z)-resveratrol
CAS number: 61434-67-1
Molecular formula: $C_{14}H_{12}O_3$
Molecular weight: 228.247 g/mol

2. HPLC analysis

Column: Agilent ZORBAX SB-C_{18} (250 × 4.6 mm, 5 μm)
Column temperature: 30℃
Detection mode: UV 286 nm
Flow rate: 1.0 mL/min
Sample dissolution: Methanol
Mobile phase: A, 0.1% phosphoric acid in water; B, acetonitrile
Gradient elution: B, 20-30%, 15 min
Injection volume: 10 μL
Run time: 25 minutes

3. Chromatogram

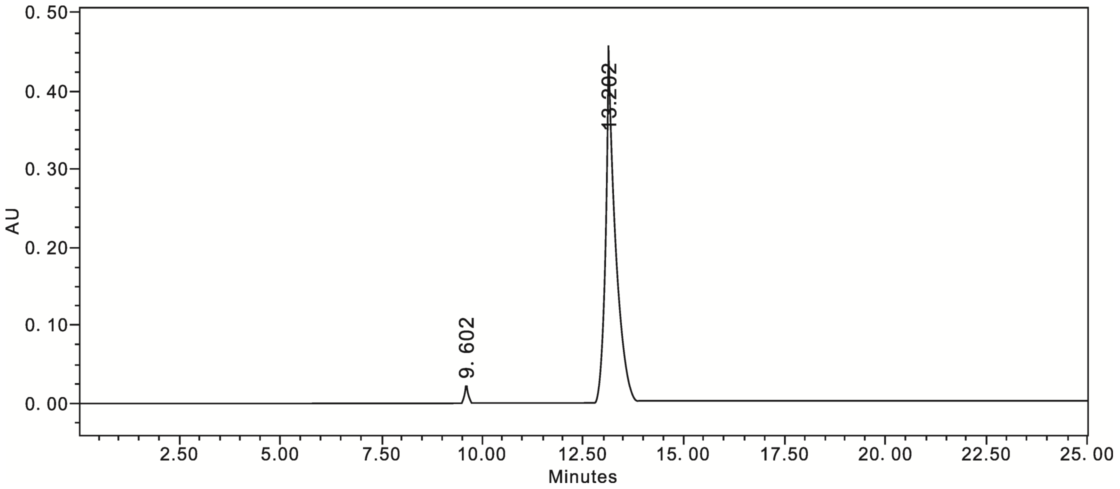

4. Analysis results

	Retention time (min)	Peak area	% area
1	9.602	174337	1.98
2	13.202	8630568	98.02

Report date: 01/03/2018

NMR data of *E*- and *Z*-resveratrol

Table S1. ^{1}H and ^{13}C chemical shifts and some coupling constants of resveratrol isomers (ppm).

Position	*E*-Resveratrol		*Z*-Resveratrol	
	^{1}H (ppm)	^{13}C (ppm)	^{1}H (ppm)	^{13}C (ppm)
1		139.2		139.1
2,6	6.37 (^{4}J = 2.2 Hz)	104.4	6.11 (^{4}J = 2.2 Hz)	106.4
3,5	9.19	158.6	9.11	158.4
4	6.10 (^{4}J = 2.2 Hz)	101.9	6.05 (^{4}J = 2.1 Hz)	101.6
1'		128.0		127.4
2',6'	7.38 (^{3}J = 8.3 Hz)	127.8	7.06 (^{3}J = 8.6 Hz)	130.0
3',5'	6.74 (^{3}J = 8.3 Hz)	115.6	6.61 (^{3}J = 8.6 Hz)	115.0
4'	9.55	157.3	9.47	156.7
1"	6.79 (^{3}J = 16.2 Hz)	125.6	6.25 (^{3}J = 12.3 Hz)	128.0
2"	6.90 (^{3}J = 16.2 Hz)	127.9	6.35 (^{3}J = 12.3 Hz)	129.4

Position refers to the numbering in Figure S1. Hydrogens 3, 5 and 4' are OH protons, all other protons are carbon-bound ones.

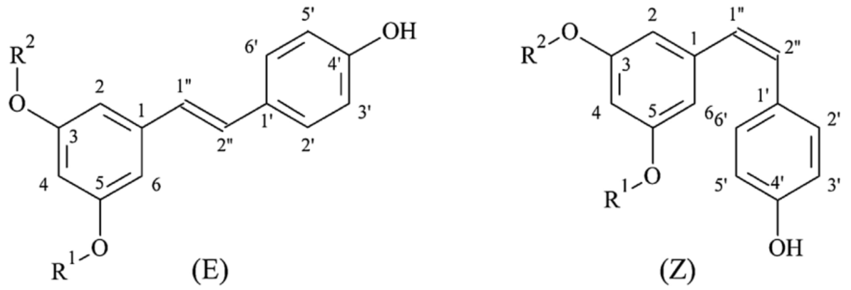

Figure S1. Structure and numbering of *E*- and *Z*-resveratrol ($R_1 = R_2 = H$).
